# Patterns of deep fine root and water utilization amongst trees, shrubs and herbs in subtropical pine plantations with seasonal droughts

**DOI:** 10.3389/fpls.2023.1275464

**Published:** 2023-09-20

**Authors:** Peipei Jiang, Jinliang Yan, Rongxin Liu, Xuejie Zhang, Shoujin Fan

**Affiliations:** ^1^ Key Lab of Plant Stress Research, College of Life Sciences, Shandong Normal University, Ji’nan, Shandong, China; ^2^ Yangji Forest Farm (Yangtianshan Provincial Nature Reserve Protection Center) of Qingzhou, Weifang, Shandong, China

**Keywords:** community structure, deep fine root, deep soil water utilization, light transmission, seasonal dynamics, soil property

## Abstract

**Introduction:**

Seasonal droughts will become more severe and frequent under the context of global climate change, this would result in significant variations in the root distribution and water utilization patterns of plants. However, research on the determining factors of deep fine root and water utilization is limited.

**Methods:**

We measured the fine root biomass and water utilization of trees, shrubs and herbs, and soil properties, light transmission, and community structure parameters in subtropical pine plantations with seasonal droughts.

**Results and Discussion:**

We found that the proportion of deep fine roots (below 1 m depth) is only 0.2-5.1%, but that of deep soil water utilization can reach 20.9-38.6% during the dry season. Trees improve deep soil water capture capacity by enhancing their dominance in occupying deep soil volume, and enhance their deep resource foraging by increasing their branching capacity of absorptive roots. Shrubs and herbs showed different strategies for deep water competition: shrubs tend to exhibit a “conservative” strategy and tend to increase individual competitiveness, while herbs exhibited an “opportunistic” strategy and tend to increase variety and quantity to adapt to competitions.

**Conclusion:**

Our results improve our understanding of different deep fine root distribution and water use strategies between overstory trees and understory vegetations, and emphasize the importance of deep fine root in drought resistance as well as the roles of deep soil water utilization in shaping community assembly.

## 1 Introduction

Under the context of global climate change, the intensity, frequency, and duration of drought are increasing, leading to changes in water utilization of plants as well as decreases in plant productivity and survival (Allen et al., 2010; Adams et al., 2017; Ripullone et al., 2020). To some extent, water uptake and utilization patterns of plants reflect the response of ecosystems to variations in environmental hydraulic status (Gulihanati et al., 2022). A better knowledge of plants’ water use is necessary to enhance our understanding of plants adaptations to changes in the forest hydrologic cycle and our predictions on variations of the community composition and function in forest ecosystems with climate drying (Ding et al., 2018; Ripullone et al., 2020).

The distribution of roots is closely associated with the water access of plants. In forest ecosystems, trees usually distribute more fine roots in deeper layer than understory shrubs and herbs, and shrubs often distributed their fine root deeper than herbs (Achat et al., 2008; Sun et al., 2015). According to the two-layer model of woody and herbs coexistence (Walker and Noy-Meir, 1982), herbs with shallower roots are competitors of shallow soil moisture, and woody plants with deeper roots could monopolize the deep soil moisture. This model has been confirmed by many studies (Le Roux et al., 1995; Moreira et al., 2000; Eggemeyer et al., 2009). Previous studies also shown that deep soil water absorbed by trees with deeper roots can be utilized by shrubs and herbs with shallower roots through hydraulic lift (Yu and D'Odorico, 2015; Barron-Gafford et al., 2021). However, some studies shown that root depth is not directly associated with water uptake depth, since the water uptake of plants depended mainly on active roots (Ehleringer and Dawson, 1992; West et al., 2007). Therefore, it is very essential to determine the relationships between root distribution and water use of plants amongst life forms. In addition, intraspecific or interspecific competition also can affect the distribution of fine root and water utilization. Also, roots of trees that grown with understory vegetations was deeper than those without competition, while roots of herbs that grown with shrubs are mostly distributed in shallow soil (Rolo and Moreno, 2012; Cardinael et al., 2015). Similarly, sea-buckthorn in a mixed forest of trees and shrubs had higher deep soil water reliance than those in pure sea-buckthorn forest during the dry season (Wu et al., 2022). Moreover, *Robinia pseudoacacia* in the Loess Plateau was found to increase the reliance of shallow and middle soil water but decrease the reliance of deep soil water as thinning intensity increased (Liu et al., 2023). Current researches mainly focus on variations of root and soil water utilization at the upper 1 m depth of the soil profile, however, the distribution patterns of fine root and soil water utilization below the 1 m depth are still unclear.

Generally, plant water utilization is related to the distribution of soil moisture with time and soil depth (Eggemeyer et al., 2009; Chen et al., 2014; Yang et al., 2015). Plants with dimorphic root systems could transfer the main water source from shallow soil to deep soil with soil drying (Quesada et al., 2008; Eggemeyer et al., 2009; Chen et al., 2014). For example, Yang et al. (2015) discovered that three coniferous plants in subtropical region with a marked seasonal dry season transferred their main water source to deep soil layer in the dry season. Also, *Vitex negundo* in the Loess Plateau was found to primarily utilize top soil water of 0-40 cm layer and gradually transferred to soil water of deeper layers as the season progressed (Wang et al., 2017). Similarly, Zhu et al. (2021) found that *R. pseudoacacia* and *Jiziphus jujuba* in the Taihang Mountains mainly used soil water of the top soil layer (0-10 cm) in the wet season and that of the deeper soil layer (30-50 cm) in the dry season. However, plants’ water use strategies can be variable due to the micro local changes in soil conditions, i.e. soil depth, stoniness, water holding capacity (Love et al., 2019; Carrière et al., 2020b). Because the water retention capacity of shallow soil was poor, plants growing on continuous dolostone outcrops and nearby thin soil layers mainly used deep soil moisture in both the wet and dry seasons (Nie et al., 2011). Similarly, woody plants with poor surface conditions (i.e. low water holding capacity) could allow their roots to utilize deep water reserves more intensively than those with better surface conditions (Carrière et al., 2020a).

Other abiotic factors (e.g. soil bulk density, rock fragment content, and light) can also affect the distribution patterns of fine roots and water use of plants. Generally, the development of root was severely restricted with the increase of soil bulk density since high mechanical could impede the elongation and proliferation of root (Pabin et al., 1998; McIvor et al., 2014). However, there are different opinions on whether the existence of rock fragment is beneficial for root growth of plant. Novák and Knava (2012) found that the existence of rock fragments can reduce soil hydraulic conductivity and water retention capacity and therefore affect the soil water availability for plants. In contrast, there are also studies showed that rock fragments can serve as reservoirs of plants and water conservation was found to be better in rocky soils under moderate water stress conditions (Danalatos et al., 1995; Tetegan et al., 2015). Moreover, light also had an important influence on the distribution of fine root, though this effect may be dependent on the availability of soil water and/or nutrients (Valladares and Pearcy, 2002; Schall et al., 2012). Generally, the amount of water that can be tapped and its transportation from roots to leaves is thought to be mainly related to transpiration without consuming metabolic energy (Ksenzhek and Volkov, 1998), which largely depends on the degree of opening and closing of stomata. However, the tension gradient caused by transpiration may be not sufficient to stimulate the transport systems of plants, and then plant would employ root pressure to pump water into xylem vessels at the cost of minimal metabolic energy consumption (Schwinning, 2010; Wu et al., 2021). Therefore, capturing and pumping from deep depth required more energy (through root pressure) than extracting shallow soil water. Therefore, low light transmittance could affect the transpiration and photosynthetic rates of plants and therefore affect the water utilization of plants (especially deep soil water utilization). However, studies on determining factors of deep soil water utilization across life forms still very lack.

In our study area, the understory vegetation species are rich and the soil heterogeneity is high, which can induce strong variations in soil conditions at very close distances (Jiang et al., 2023). In turn, this might affect the distribution of fine root and water utilization for vegetations (Gargiulo et al., 2016; Love et al., 2019; Carrière et al., 2020a). This provides a suitable opportunity to research the distribution patterns of fine root and water utilization as well as their determining factors. Therefore, we measured the fine root distribution and water use of trees, shrubs, and herbs, community structure parameters (stem density of trees, and the dominance, richness and evenness of understory shrubs and herbs), light transmission, and soil properties (soil bulk density, pH, water content, rock fragment content, total carbon and nitrogen concentration) in subtropical pine plantations with seasonal droughts. The main aims of our study were: (i) to compare the deep fine root and water utilization allocation strategies amongst trees, shrubs and herbs; (ii) to determine the allocation of deep fine root and their determinants (including community structure parameters, soil properties, and light transmission) amongst trees, shrubs and herbs; (iii) to examine the deep soil water utilization strategy and their determinants (including fine root biomass, community structure parameters, soil properties, and light transmission) amongst life forms.

## 2 Materials and methods

### 2.1 Site description

**Figure 1:**
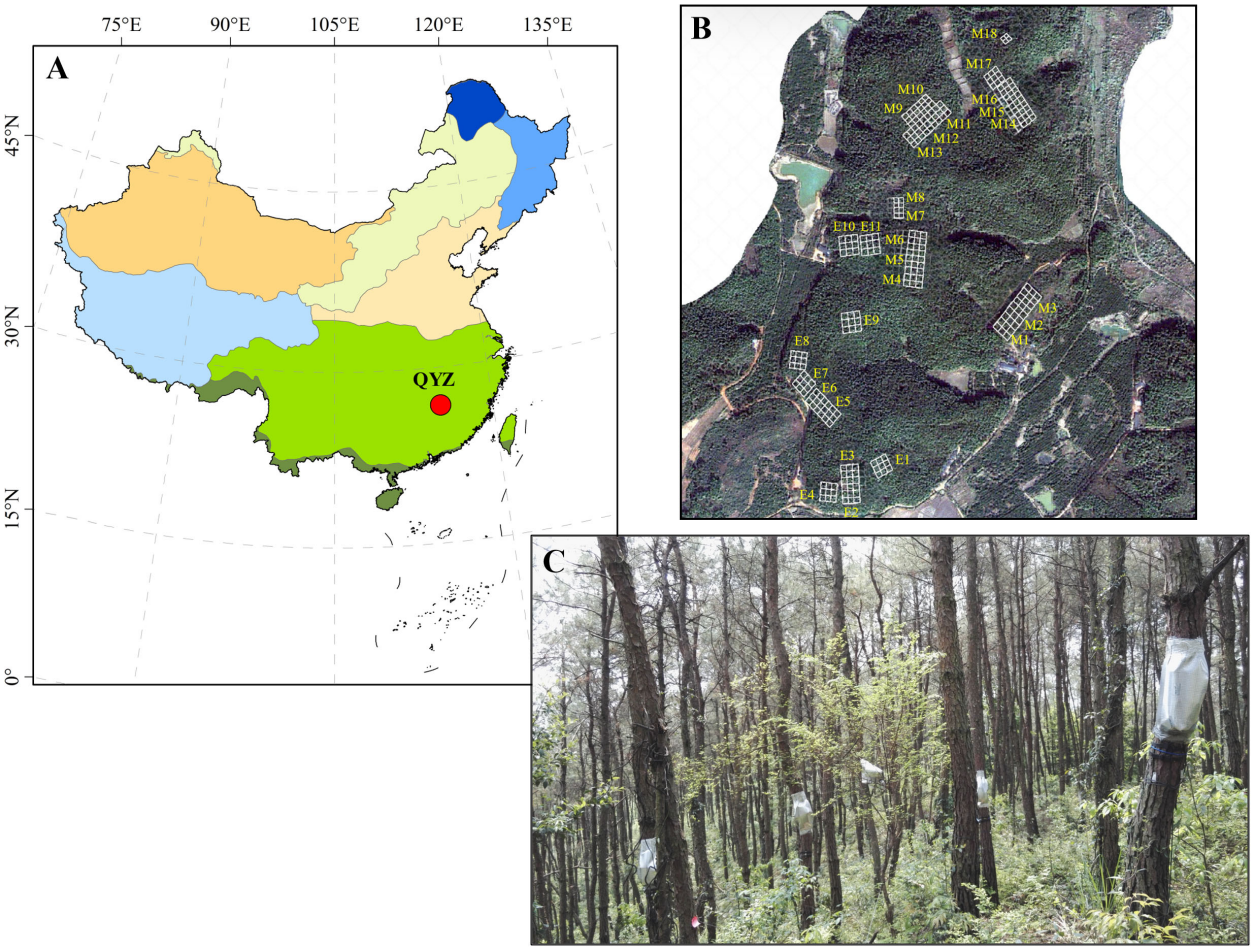
Location of study area (in red circle, **A**), and distribution **(B)** and vegetation picture **(C)** of the sampling plots. In **(B)** M1-M18 represent the 18 plots of *P. massoniana* plantations and E1-E11 represent the 11 plots of *P. elliottii* plantations.

This research was conducted at the Qianyanzhou Forest Ecosystem Research Station of the Chinese Academy of Sciences (QYZ for abbreviation; 26°44′39′′N, 115°03′33′′E), which is located in the Jiangxi Province of southeastern China (red circle in **Figure 1A**). Due to the uneven distribution of rainfall, seasonal droughts occur frequently in this region. The mean annual temperature and precipitation are 18.0°C and 1509.0 mm, respectively. The soil is an iron-rich red soil classified as Typic Dystrudept and Udept Inceptisols by the USDA soil taxonomic system. The vegetation mainly consists of *Pinus massoniana* and *P. elliottii* plantations which were planted in 1983, and the vegetation picture was shown in **Figure 1C**.

### 2.2 Experimental design and stand structures

A total of 29 plots (30 × 30 m) were established in the pine plantations, including 18 plots of *P. massoniana* and 11 plots of *P. elliottii*. The distribution of these plots was shown in **Figure 1B**. The dominant shrub species across 29 plots were *Loropetalum chinensis*, *Adinandra millettii*, *Camellia oleifera*, *Rhus chinensis*, *Eurya muricata* and *Rhaphiolepis indica*. The dominant herb species across 29 plots were *Dryopteris atrata*, *Woodwardia japonica*, *Dicranopteris dichotoma*, *Morinda umbellate*, and *Lophatherum gracile*.

The community structure was investigated in August 2015. Each plot was divided into nine quadrats (10 × 10 m) and then taken photos below the canopy. Light transmission was then determined by Side look and Gap Light Analyzer (Frazer et al., 1999). In each plot, the diameter at breast height (DBH) of each tree was measured. Three shrub subplots of 5 × 5 m were set along the diagonals within each tree plot. Then, the basal diameters, heights, and number of bushes of all shrubs in each subplot were measured. One herb quadrat of 1 × 1 m was set within each shrub subplot, and the heights and coverages of all herbs were then measured in each quadrat.

### 2.3 Soil properties and fine-root distributions

Soil cores of 10 cm diameter were sampled every 20 cm at the up-, mid-, and down slopes from soil surface to 200 cm depth in each plot. Living fine roots were carefully picked out and gently washed with running water. Living fine roots were then sorted to trees, shrubs, and herbs, according to their contrasting traits in terms of morphological structure, color, and mechanics (e.g. rigid or soft, rough or smooth). More details can be seen in Jiang et al. (2018a). The fine roots were classified using an order-based approach described by Pregitzer et al. (2002). Fine roots of woody species (trees and shrubs) were then divided into absorptive (1-3 order) and transport (4-5 order) categories, while those of herbs were not distinguish between the absorptive and transport categories.

After root extraction, rock fragments (> 2 mm) were picked up from the soil samples, and then air-dried and weighed. The rock fragment content of each soil sample was determined by using a stone density of 2.65 g cm^-3^ (Diochon et al., 2009). Due to the rocky properties of soil in our study area, the use of standard soil core method could not accurately determine the soil bulk density, and therefore the differences between volumes of soil cores and stones were applied to determine the soil bulk density (Klinka et al., 1981; Diochon et al., 2009). Soil pH was determined in a 1:2.5 mixture of air-dried soil/distilled water mixture by using an electrode pH meter (S40, Mettler Toledo, Switzerland). Total carbon and nitrogen contents of the surface layer (0-20 cm) were measured by an elemental analyser (vario MACRO cube, Germany).

### 2.4 Soil water utilization of plants

Plant samples for water isotope analysis was collected during mid-morning in both the dry season (August 2016) and the wet season (April 2017). For trees, stems from two to three sample trees of middle diameter were collected in each plot, and phloem tissue was peeled off to avoid potential contamination (Querejeta et al., 2007). For shrubs and herbs, ten shrub species and three fern species were sampled in each plot, with the average coverage of the collected species relative to the total coverage of all shrubs or herbs was 63.6 ± 3.3% and 75.3 ± 5.8% across 29 plots, respectively. The water use of these collected shrubs and herbs was used for representing the overall water use of the shrub and herb layers. For shrubs, green tissues were removed from the branches to avoid contamination (Ehleringer and Dawson, 1992). Meanwhile, the basal diameter of shrub individuals was controlled between 10 and 25 mm to avoid the potential effects of plant age on water absorption. For herbs, the thick and fleshy root crowns were collected, since this part had the most stable water isotopic ratios (Barnard et al., 2006). Soil samples were collected at depth of 0-20, 20-60, 60-100, 100-150, 150-200 cm in each plot. Then, each soil sample was separated into two subsamples: one subsample was used for water extraction, and the other subsample was used for measuring the soil water content. Soil water content was then measured by dividing fresh weight minus dry weight by dry weight.

We extracted water from plant and soil samples through an automatic low-temperature vacuum distilled water extraction system (Li-2100, Beijing, China). The extraction time for plant and soil sample was 3 h and 2.5 h, respectively. We analyzed the water isotope ratio of water samples using a liquid water isotope analyzer (912-0050, LGR, California, USA). The IsoSource model was applied to calculate the relative contribution of soil water at different depths to plant (Phillips et al., 2005). See Jiang et al. (2020b) for details. Because the accuracy in hydrogen isotopes analysis was relatively lower than that of oxygen stable isotopes when using the Isotopic Ratio Infrared Spectroscopy (IRIS) method (Wen et al., 2012), δ^18^O was shown to be more sensitive than δD in evaluating water uptake of plant and therefore δ^18^O was used to detect the water uptake depths of plants.

### 2.5 Data analysis

Due to the 0-100 cm soil depth accounts for over 90% of the total fine root biomass of the entire 0-200 cm soil profile in all life forms and the δ^18^O value of the upper 100 cm soil was different from the below 100 cm depth (**Figures S1**, **S2**), the 100-200 cm depth was defined as the deep soil layer. Then, fine root biomass in the deep soil layer was determined by the sum of fine root biomass below the 100 cm depth of the entire 0-200 cm soil profile. The deep soil water utilization was determined by the sum of soil water proportions in 100-150 and 150-200 cm depth. Since the P. massoniana and P. elliottii plantations had the similar root distribution and water utilization allocation patterns (Yang et al., 2015, Yang et al., 2017; Jiang et al., 2020b), we combined the data from the two pine plantations. For trees, the seasonal plasticity of deep soil water utilization (DWP) was calculated as:


(1) $$\text{DWP}=\frac{2\times \left(\text{DDW}-\text{WDW}\right)}{\text{DDW}+\text{WDW}}$$

where DDW is the deep soil water utilization during the dry season and WDW is the deep soil water utilization during the wet season (Padilla et al., 2007; Jiang et al., 2020a; Jiang et al., 2020b). For shrubs and herbs, the community-weighted mean DDW in each plot was calculated. First, dividing the number of individuals of each species by the total number of shrub (or herb) species to determine the weight of each species in each subplot, and then calculate the weight of each species in each plot as the mean value of the three subplots. Second, the community-weighted mean DDW of each plot was calculated as the sum of the product of the average weight of each species and its DDW. Then, the community-weighted mean DDW of shrubs and herbs was used to calculate the DWP according to equation (1). The biodiversity index of each plot was determined by “Biodixcel.xlsx” in Microsoft Office Excel 2007 (Kong et al., 2012).

To test the differences of fine root biomass and water source in deep soil layer amongst life forms, a one-way analysis of variance (ANOVA) was conducted in SPSS (2010, ver.19.0; SPSS Inc., USA). To test the differences between trees and shrubs (or between seasons), the independent sample T test was applied in SPSS. To analyse the relationships between the fine root biomass and water utilization in the deep soil layer and the abiotic and biotic factors, the RDA-ordination biplot was carried out in the CANOCO software (ver.5.0, Ithaca, NY, USA). Significance was defined at the 0.05 level.

## 3 Results

### 3.1 Deep fine root biomass and water utilization amongst life forms

**Figure 2:**
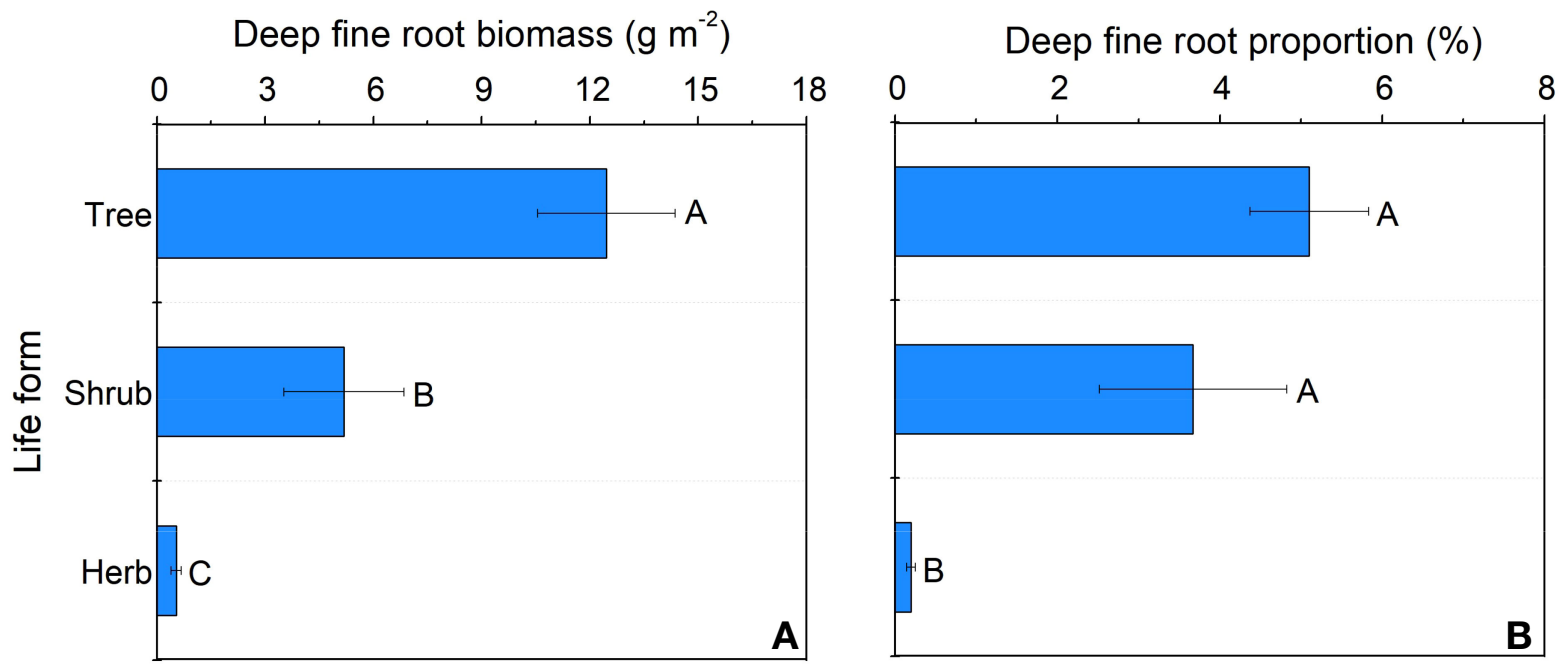
Deep fine root biomass **(A)** and proportion **(B)** for different life forms. Different letters represent significant differences in deep fine root biomass or proportion across life forms.

**Figure 3:**
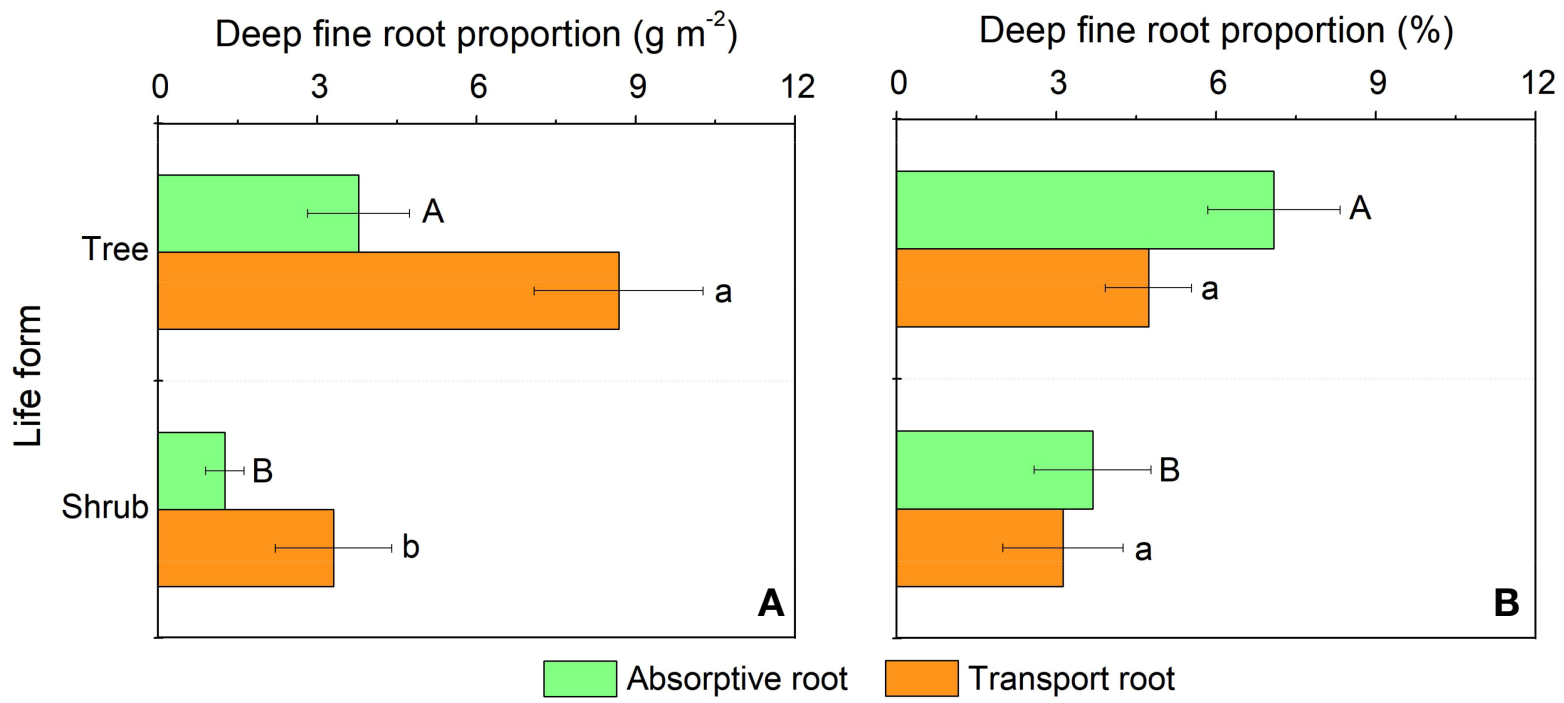
Biomass **(A)** and proportion **(B)** of the absorptive and transport fine root biomass for trees and shrubs in deep soil layer. Different uppercase and lowercase letters represent significant differences in deep fine root proportion for absorptive and transport fine root between trees and shrubs, respectively.

**Figure 4:**
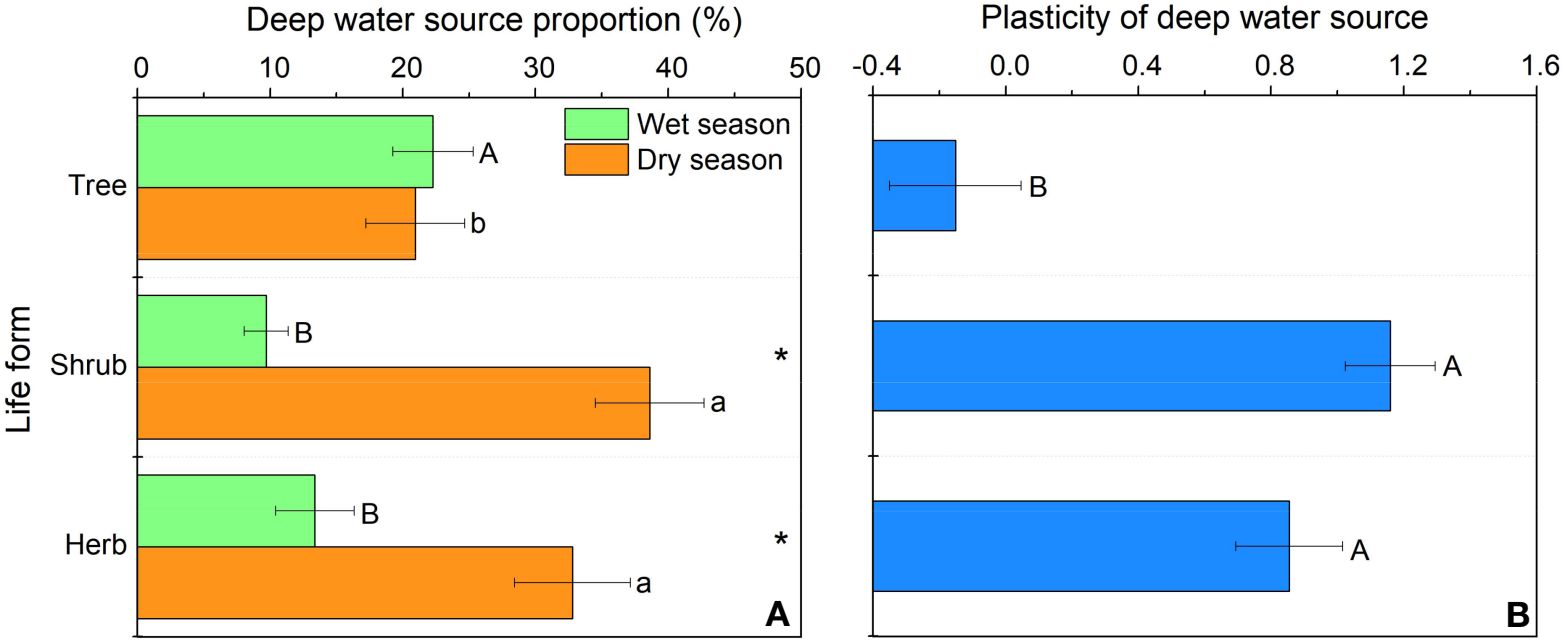
Deep soil water utilization in the wet and dry season **(A)** and its seasonal plasticity **(B)** for different life forms. In **(A)** different uppercase and lowercase letters represent significant differences in deep soil water utilization during the wet or dry season across life forms, respectively; in **(B)** different letters represent significant differences in seasonal plasticity of deep soil water use across life forms.

In our study, trees and understory vegetations showed contrast deep fine allocation and water use strategies (**Figures 2**, **3**). We found trees and shrubs distributed more fine roots than herbs in deep soil layer (**Figure 2**). The fine root biomass of trees was significantly higher than that of shrubs, however, the differences in deep fine root proportion between them was not significant. Trees tended to distribute more absorptive fine roots than shrubs in deep soil, however, the deep transport fine root biomass between trees and shrubs didn’t show significant differences (**Figure 3**). Trees have a significant deep soil water reliance in both the wet and dry seasons, while shrubs and herbs had a lower deep soil water reliance during the wet season and a higher deep soil water reliance during the dry season (**Figures 4**, **S3**). Therefore, trees showed a smaller seasonal plasticity of deep soil water reliance than shrubs and herbs (**Figure 4**).

### 3.2 Determining factors of fine root and water utilization in the deep soil layer amongst life forms

**Figure 5:**
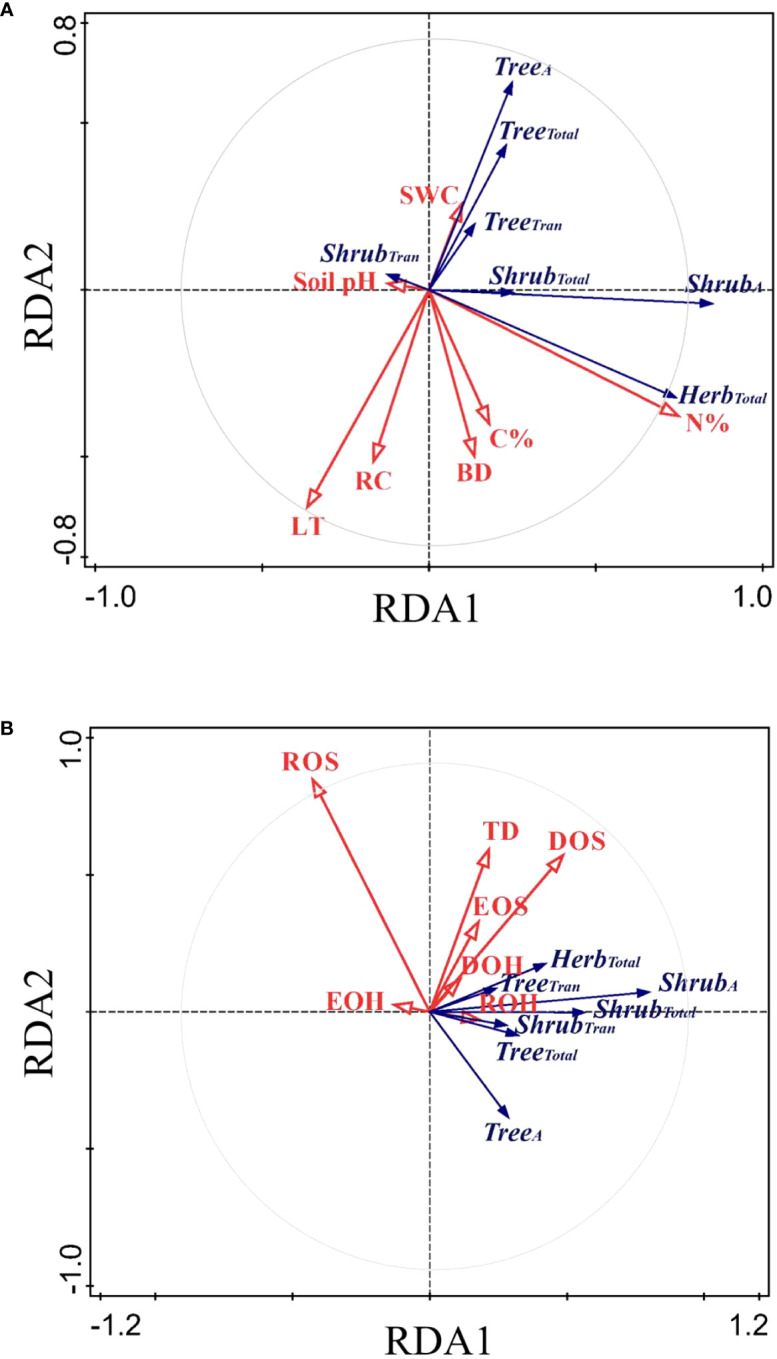
RDA-ordination biplot for the fine root biomass of deep soil layer amongst life forms and abiotic factors **(A)** and biotic factors **(B)**. Tree_A_/Shrub_A_, absorptive fine root biomass of trees/shrubs; Tree_Tran_/Shrub_Tran_, transport fine root biomass of trees/shrubs; Tree_Total_/Shrub_Total_/Herb_Total_, total fine root biomass of trees/shrubs/herbs; LT, light transmission; C%, total soil carbon content in subsoil layer; N%, total nitrogen content in subsoil layer; RC, rock fragment content in deep soil layer; BD, soil bulk density in deep soil layer; SWC, soil water content in deep soil layer; pH, soil pH in deep soil layer. The same below.

**Figure 6:**
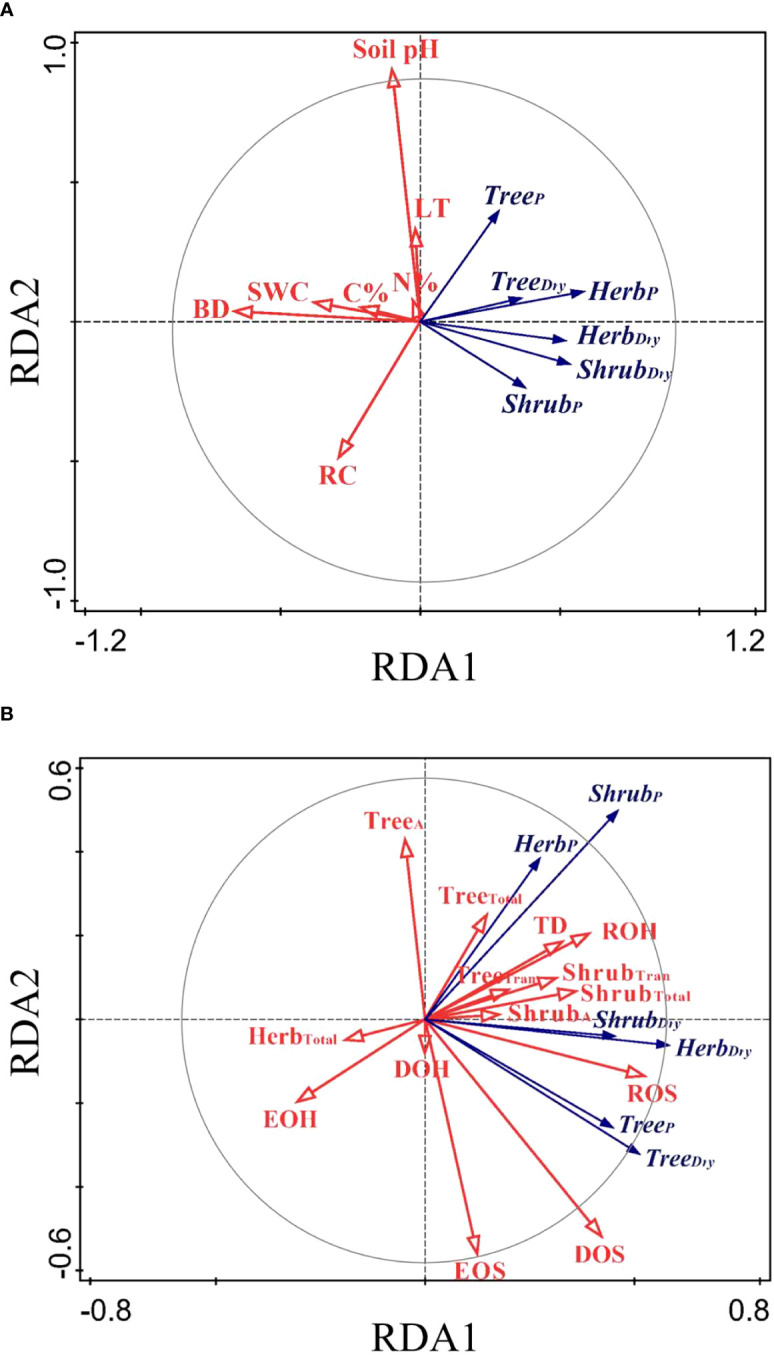
RDA-ordination biplot for deep soil water utilization and its seasonal plasticity amongst life forms and abiotic factors **(A)** and biotic factors **(B)**. Tree_Dry_/Shrub_Dry_/Herb_Dry_, deep soil water utilization of trees/shrubs/herbs in the dry season; Tree_P_/Shrub_P_/Herb_P_, seasonal plasticity in deep soil water utilization of trees/shrubs/herbs; TD, tree density; DOS/DOH, dominance of shrubs/herbs; ROS/ROH, richness of shrubs/herbs; EOS/EOH, evenness of shrubs/herbs.

The RDA ordination biplot displayed that the major determining abiotic and biotic factors of deep fine root and water utilization were different (**Figures 5**, **6**). For the deep fine root biomass, the C%, N%, light transmission, and rock fragment content were the major abiotic factors (**Figure 5A**), which contributed 35.7%, 25.3%, 17.0%, and 11.3% variations in these factors; the dominance and richness of shrubs were the major biotic factors (**Figure 5B**), which contributed 33.8% and 23.9% variations in these factors, respectively. For details, C% and N% displayed a predominantly positive relationship with total and absorptive fine root biomass of shrubs and herbs, while light transmission showed a predominantly negative relationship with fine root biomass of trees; the dominance of shrubs displayed a positive relationship with fine root biomass of shrubs and herbs, while the richness of shrubs was negatively related to total and absorptive fine root biomass of trees. For the deep soil water utilization in the dry season and its plasticity between seasons, the soil bulk density and water content were the major abiotic factors (**Figure 6A**), which contributed 32.7% and 26.2% variations in these factors; the dominance and evenness of shrubs, and the richness of herbs were the major biotic factors (**Figure 6B**), which contributed 16.0%, 13.3% and 12.5% variations in these factors. For details, soil bulk density and water content were negatively correlated with deep soil water utilization in the dry season and its plasticity between seasons amongst life forms; dominance and evenness of shrubs was positively related to deep soil water utilization of trees and its seasonal plasticity, while the richness of herbs was positively associated with the deep soil water utilization of shrubs as well as its seasonal plasticity (**Table S1**).

## 4 Discussion

### 4.1 Deep fine root biomass and water utilization amongst life forms

We found trees and shrubs distributed more fine roots than herbs in deep soil. Though the proportion of total deep fine root biomass in trees and shrubs was not significant, trees had higher proportion of absorptive fine root biomass than shrubs in deep soil. Generally, absorptive roots can make more efficient use of soil resources due to the thinner diameter and thus occupying a dominant position in total length and surface area of root (Guo et al., 2004). That is, trees can effectively improve deep soil nutrient absorption efficiency by increasing root branching capacity (increase the proportion of absorptive roots) and thus adapt to the limited soil conditions (Liu et al., 2015; Li et al., 2017). This might be a key strategy for overstory trees to avoid competing with understory vegetations for limited surface soil resources. Moreover, the proportion of deep fine root biomass (below 1 m depth) is only 0.2-5.1%, but their proportion of deep soil water utilization can reach 20.9-38.6% in the dry season, which highlight the importance of deep fine root in plant drought resistance.

In our study, we found that trees and understory vegetations had contrast deep water use strategies. For details, trees have a significant deep soil water reliance throughout the year (especially with the addition of water source from 60-100 cm soil layer; **Figures 4**, **S3**), and thus had low seasonal plasticity of deep soil water use. In contrast, shrubs and herbs are less dependent on deep soil moisture during the wet season but highly dependent on deep soil moisture during the dry season, and thus had high plasticity of deep soil water reliance. This might be because trees have more fine roots distributed in deep soil layer than understory vegetations, and fine roots in the deep soil would increase the diameter of stele and xylem conduits to and thereby minimizing flow resistance and maximizing water absorption efficiency (Pate et al., 1995; Pate et al., 1998; McElrone et al., 2004). Moreover, trees tend to have higher proportions of absorptive fine roots in deep soil layer than those of shrubs. Considering that the deep soil is less fertile than the shallow soil (Jobbagy and Jackson, 2001; Jobbagy and Jackson, 2004), trees can improve their deep soil nutrient absorption efficiency by increasing their branching capacity of absorptive fine roots in the deep soil. Compared with trees, understory vegetations had greater seasonal plasticity in deep soil water utilization. This may be due to the fact that the volume of trees was larger and therefore they are more resistant to droughts than understory vegetations. Since the water storage capacity in stems of trees was larger and thus could more efficiently buffer the daily fluctuation and decrease of leaf water potential with soil drying (Chapotin et al., 2006; Oliva Carrasco et al., 2015), and their large horizontal area for root foraging can to some extent compensate for the decrease in shallow soil moisture content (Meinzer et al., 1999). Second, the seasonal plasticity of shrubs and herbs was closely correlated with fine root biomass of trees and shrubs (**Figure 6**), which indicated that the important role of hydraulic lift. Also, the high deep water use plasticity of shrubs and herbs suggests that they are good at utilizing shallower soil moisture that is briefly replenished by dry season precipitation, while trees tend to rely on deeper soil moisture which is more stable over time (Cui et al., 2017).

### 4.2 Determining factors of fine root and water utilization in deep soil layer amongst life forms

We found that the distribution of deep fine root biomass was closely associated with soil nutrient content. This may be because when the availability of soil resources increases, the carbon allocated to the belowground part would increase, and fine roots can increase the absorption capacity of water and nutrients in soil by changing root morphological characteristics (Curt and Prévosto, 2003). Generally, the fine root biomass of understory vegetations (especially the shallow layer) increased with light transmission (Matjaž and Primož, 2010; Noguchi et al., 2021), and thus the competition between overstorey and understory vegetations would intensify. Therefore, we discovered that the fine root biomass of trees in deep soil layer was negatively related to the light transmission. We also found that the fine root biomass of trees displayed a negative correlation with rock fragment content, which may be explained by that the existence of rock fragments could reduce soil hydraulic conductivity and water retention capacity and therefore decrease the soil water availability for plants (Novák and Knava, 2012). However, previous studies also shown that rock fragments can serve as reservoirs for plants and water conservation was found to be better in rocky soils under moderate water stress conditions (Danalatos et al., 1995; Tetegan et al., 2015). The reason for these contrast results may be that rock fragments had a positive effect on plant growth and water consumption below a certain threshold, and therefore it is necessary to optimize the rock fragment content when evaluating water relations of plants in rocky ecosystems (Mi et al., 2016). In our study, the deep soil water utilization of plants increased with the decrease of deep soil water content. Similarly, the shallow soil water content was found to remain low and the deep soil moisture showed a downward trend in previous studies, which indicated that plants increase the deep soil water utilization to ensure growth in the dry season (Jia and Shao, 2014; Cao et al., 2018; Li et al., 2021). Also, deep soil bulk density was negatively related to the deep soil water utilization of plants, which was mainly due to high mechanical could restrict the elongation and proliferation of root (Szota et al., 2007; McIvor et al., 2014; Miyatani et al., 2016).

Our results showed that the main biotic determining factors of the distribution of deep fine root biomass were the dominance and richness of shrubs (**Figure 5B**). In our study, the biomass of fine roots of shrubs displayed a positive correlation with shrub dominance. However, the absorptive and total fine root biomass of trees were negatively related to the richness of shrubs, indicating that deep fine root biomass of trees decreased due to intense competition with shrubs. Our results showed that the main biotic factors affecting the distribution of deep soil water utilization were the dominance and evenness of shrubs, and the richness of herbs (**Figure 6B**). Also, deep soil water utilization of trees could increase the dominance of shrubs, and that of shrubs could increase the richness of herbs in our study (**Figure 6**, **Table S1**). Similarly, previous studies showed that trees likely influence local resource availability (e.g. light, soil) and serve as a biotic filter in the understory community assembly (Jiang et al., 2018b; Luo et al., 2019). This might because the plots with higher deep soil water utilization of trees increased the probability of hydraulic uplift, and therefore the companion shrubs could obtain more water resource, thus increasing the dominance of shrubs. Similarly, the plots with higher deep soil water utilization of shrubs could also enhance the probability of the deep soil water being lifted and released into the surface soil, which could provide more water resources for shallow-rooted herb plants. This induced the enrichment of shallow-rooted herbs in the areas of hydraulic uplift to some extent, thus improving the richness of herbs in these regions (Maestre et al., 2003; Šenfeldr et al., 2016). Moreover, the plots with deep soil water utilization of trees (or shrubs) meant that shallow soil water consumption was more severe and the competition of water resource between different life forms was more intense. Therefore, the positive relationships between deep soil water utilization of trees and the dominance of shrubs and between deep soil water utilization of shrubs and the richness of herbs indicated that shrubs and herbs exhibited different “strategy” in response to competition. For details, shrubs exhibited a “conservative strategy” and tend to increase individual competitiveness and could maximize their resource acquisition in habitats with little disturbance. In contrast, herbs exhibited an “opportunistic strategy” and tend to increase variety and quantity to adapt to the competition and could survive in habitats with frequent disturbance. Our results highlighted the roles of deep soil water utilization in shaping community assembly as well as the necessary of linking biodiversity to ecosystem function (Schwartz et al., 2000; Dudgeon, 2010).

In our study, the explanation degree of RDA ordination biplot was only *c*. 30%, which was relatively low. This may be explained by that the traits we didn’t measure (e.g. photosynthetic related properties, traits related to plant’s water status), could further play important roles in determining the deep fine root and water utilization and thus the community assembly. Compared with shallow root, deep fine roots have larger diameters and higher tissue density and thus require more carbon investment (Prieto et al., 2015; Fort et al., 2017). Therefore, deep roots should be more closely related to physiological functions such as photosynthesis. Also, plant deep soil water utilization should be closely correlated with stomatal conductance and transpiration rate, since capturing and pumping from deeper soil required more energy (through root pressure) than extracting shallow soil water (Ksenzhek and Volkov, 1998; Wu et al., 2021). Similarly, plant water uptake was found to be closely associated with stomatal conductance, transpiration rate, and stomatal density (Romero-Aranda et al., 2001; Hepworth et al., 2016). This can also explain the positive correlation between light transmission and deep soil water utilization. Previous studies proposed that root hydraulics (instead of root length) could determine the water consumption of crops (Vadez, 2014; Cai et al., 2022), since root conductance synthesizes the effects of the architecture, length, and anatomy of root (Doussan et al., 2006; Strock et al., 2021). Similarly, our previous studies at the species level in the study area showed a positive correlation between deep water utilization and stem hydraulic conductance (Jiang et al., 2020a). Also, recent studies have showed that water uptake depth was coordinated with leaf economic spectrum in water-limited ecosystems (Pivovaroff et al., 2021; Illuminati et al., 2022). Therefore, future studies on plant water use should also focus on plant physiological properties (e.g photosynthetic related properties, hydraulic related properties) and economic spectrum traits.

## 5 Conclusion

Our results provide new insights into the different deep fine root distribution and water use strategies between trees and understory vegetations and improve our understandings of plants adaptations to variations in the forest hydrologic cycle and thus the community assembly processes. Although the proportion of deep fine roots (below 1 m depth) is only 0.2-5.1%, their proportion of deep soil utilization can reach 20.9-38.6% in the dry season, which highlight the roles of deep fine root in drought resistance. Compared with understory vegetations, trees had a significant deep soil water reliance throughout the year and distributed a higher proportion of absorptive roots in deep soil layer. This indicated that trees could improve deep soil water capture capacity by enhancing their dominance in occupying deep soil volume, and enhance their deep resource foraging by increasing their branching capacity of absorptive roots. Moreover, shrubs and herbs showed different strategies for deep water competition: shrubs and herbs exhibited different “strategy” in response to competition: shrubs exhibited a “conservative” strategy and tend to increase individual competitiveness, while herbs exhibited an “opportunistic” strategy and tend to increase variety and quantity to adapt to the competition. Due to the low interpretation rate of RDA ordination biplot, we assumed that traits we didn’t measure might further play important roles in determining the deep fine root and water utilization and thus the community assembly. Future studies on deep fine root and water use patterns should also focus on plant physiological properties (e.g photosynthetic related properties, hydraulic related properties) and economic spectrum traits.

## Data availability statement

The original contributions presented in the study are included in the article/**Supplementary Material**. Further inquiries can be directed to the corresponding authors.

## Author contributions

PJ: Funding acquisition, Investigation, Methodology, Software, Writing – original draft, Writing – review & editing. JY: Investigation, Writing – review & editing. RL: Investigation, Writing – review & editing. XZ: Methodology, Writing – review & editing. SF: Funding acquisition, Methodology, Writing – review & editing.

## References

[B1] Achat D. L. Bakker M. R. Trichet P. (2008). Rooting patterns and fine root biomass of *Pinus pinaster* assessed by trench wall and core methods. J. For. Res. 13, 165–175. doi: 10.1007/s10310-008-0071-y

[B2] Adams H. D. Zeppel M. J. B. Anderegg W. R. L. Hartmann H. Landhäusser S. M. Tissue D. T.. (2017). A multi-species synthesis of physiological mechanisms in drought-induced tree mortality. Nat. Ecol. Evol. 1, 1285–1291. doi: 10.1038/s41559-017-0248-x 29046541

[B3] Allen C. D. Macalady A. K. Chenchouni H. Bachelet D. McDowell N. Vennetier M.. (2010). A global overview of drought and heat-induced tree mortality reveals emerging climate change risks for forests. *Forest Ecol* . Manag. 259, 660–684. doi: 10.1016/j.foreco.2009.09.001

[B4] Barnard R. L. Bello F. D. Gilgen A. K. Buchmann N. (2006). The δ^18^O of root crown water best reflects source water δ^18^O in different types of herbaceous species. Rapid Commun. Mass Sp. 20, 3799–3802. doi: 10.1002/rcm.2778 17120278

[B5] Barron-Gafford G. A. Knowles J. F. Sanchez-Cañete E. P. Minor R. L. Lee E. Sutter L. Scott R. L.. (2021). Hydraulic redistribution buffers climate variability and regulates grass-tree interactions in a semiarid riparian savanna. Ecohydrology 14, e2271. doi: 10.1002/eco.2271

[B6] Cai G. Ahmed M. A. Abdalla M. Carminati A. (2022). Root hydraulic phenotypes impacting water uptake in drying soils. Plant Cell Environ. 45, 650–663. doi: 10.1111/pce.14259 35037263 PMC9303794

[B7] Cao R. Jia X. Huang L. Zhu Y. Wu L. Shao M. (2018). Deep soil water storage varies with vegetation type and rainfall amount in the Loess Plateau of China. Sci. Rep. 8, 12346. doi: 10.1038/s41598-018-30850-7 30120347 PMC6098091

[B8] Cardinael R. Mao Z. Prieto I. Stokes A. Dupraz C. Kim J. H.. (2015). Competition with winter crops induces deeper rooting of walnut trees in a Mediterranean alley cropping agroforestry system. Plant Soil 391, 219–235. doi: 10.1007/s11104-015-2422-8

[B9] Carrière S. D. Ruffault J. Cakpo C. B. Olioso A. Doussan C. Simioni G.. (2020a). Intra-specific variability in deep water extraction between trees growing on a Mediterranean karst. J. Hydrol. 590, 125428. doi: 10.1016/j.jhydrol.2020.125428

[B10] Carrière S. D. Ruffault J. Pimont F. Doussan C. Simioni G. Chalikakis K.. (2020b). Impact of local soil and subsoil conditions on inter-individual variations in tree responses to drought: insights from Electrical Resistivity Tomography. Sci. Total Environ. 698, 134247. doi: 10.1016/j.scitotenv.2019.134247 31494427

[B11] Chapotin S. M. Razanameharizaka J. H. Holbrook N. M. (2006). Water relations of baobab trees (*Adansonia* spp. L.) during the rainy season: does stem water buffer daily water deficits? Plant Cell Environ. 29, 1021–1032. doi: 10.1111/j.1365-3040.2005.01456.x 17080930

[B12] Chen Y.-J. Cao K.-F. Schnitzer S. A. Fan Z.-X. Zhang J.-L. Bongers F. (2014). Water-use advantage for lianas over trees in tropical seasonal forests. New Phytol. 205, 128–136. doi: 10.1111/nph.13036 25264136

[B13] Cui Y.-Q. Ma J.-Y. Feng Q. Sun J.-H. Sun W. (2017). Water sources and water-use efficiency of desert plants in different habitats in Dunhuang, NW China. Ecol. Res. 32, 243–258. doi: 10.1007/s11284-017-1433-8

[B14] Curt T. Prévosto B. (2003). Rooting strategy of naturally regenerated beech in silver birch and Scots pine woodlands. Plant Soil 255, 265–279. doi: 10.1023/A:1026132021506

[B15] Danalatos N. G. Kosmas C. S. Moustakas N. C. Yassouglou N. (1995). Rock fragments II Their impact on soil physical properties and biomass production under Mediterranean conditions. Soil Use Manage. 11, 121–126. doi: 10.1016/0148-9062(96)85003-0

[B16] Ding Y. Nie Y. Schwinning S. Chen H. Yang J. Zhang W.. (2018). A novel approach for estimating groundwater use by plants in rock-dominated habitats. J. Hydrol. 565, 760–769. doi: 10.1016/j.jhydrol.2018.08.033

[B17] Diochon A. Kellman L. Beltrami H. (2009). Looking deeper: an investigation of soil carbon losses following harvesting from a managed northeastern red spruce (*Picea rubens Sarg.*) forest chronosequence. For. Ecol. Manage. 257, 413–420. doi: 10.1016/j.foreco.2008.09.015

[B18] Doussan C. Pierret A. Garrigues E. Pagès L. (2006). Water uptake by plant roots: ii—modelling of water transfer in the soil root-system with explicit account of flow within the root system—comparison with experiments. Plant Soil 283, 99–117. doi: 10.1007/s11104-004-7904-z

[B19] Dudgeon D. (2010). Prospects for sustaining freshwater biodiversity in the 21st century: linking ecosystem structure and function. Curr. Opin. Env. Sust. 2, 422–430. doi: 10.1016/j.cosust.2010.09.001

[B20] Eggemeyer K. D. Awada T. Harvey F. E. Wedin D. A. Zhou X. H. Zanner C. W. (2009). Seasonal changes in depth of water uptake for encroaching trees *Juniperus virginiana* and *Pinus ponderosa* and two dominant C_4_ grasses in a semiarid grassland. Tree Physiol. 29, 157–169. doi: 10.1093/treephys/tpn019 19203941

[B21] Ehleringer J. R. Dawson T. E. (1992). Water uptake by plants: perspectives from stable isotope composition. Plant Cell Environ. 15, 1073–1082. doi: 10.1111/j.1365-3040.1992.tb01657.x

[B22] Fort F. Volaire F. Guilioni L. Barkaoui K. Navas M. L. Roumet C. (2017). Root traits are related to plant water-use among rangeland Mediterranean species. Funct. Ecol. 31, 1700–1709. doi: 10.1111/1365-2435.12888

[B23] Frazer G. W. Canham C. D. Lertzman K. P. (1999). Gap light analyzer (GLA), version 2.0: imaging software to extract canopy structure and gap light transmission indices from true-color fisheye photographs (Millbrook, New York: Copyright 1999: Simon Fraser University, Burnaby, BC, and the Institute of Ecosystem Studies). Available at: http://www.rem.sfu.ca/forestry/index.htm http://www.ecostudies.org.

[B24] Gargiulo L. Mele G. Terribile F. (2016). Effect of rock fragments on soil porosity: a laboratory experiment with two physically degraded soils. Eur. J. Soil Sci. 67, 597–604. doi: 10.1111/ejss.12370

[B25] Gulihanati B. Chang L. Li H. Bahejiavinaer T. Zhang Y. (2022). Differences in water sources of four main shrubs of Tianshan Mountains in summer. Acta Ecol. Sin. 42, 5471–5480. doi: 10.5846/stxb202102030366

[B26] Guo D. Mitchell R. J. Hendricks J. J. (2004). Fine root branch orders respond differentially to carbon source-sink manipulations in along leaf pine forest. Oecologia 140, 450–457. doi: 10.1007/s00442-004-1596-1 15179577

[B27] Hepworth C. Turner C. Landim M. G. Cameron D. Gray J. E. (2016). Balancing water uptake and loss through the coordinated regulation of stomatal and root development. PLoS One 11, e0156930. doi: 10.1371/journal.pone.0156930 27275842 PMC4898744

[B28] Illuminati A. Querejeta J. I. Pías B. Escudero A. Matesanz S. (2022). Coordination between water uptake depth and the leaf economic spectrum in a Mediterranean shrubland. J. Ecol. 110, 1844–1856. doi: 10.1111/1365-2745.13909

[B29] Jia Y. Shao M. (2014). Dynamics of deep soil moisture in response to vegetational restoration on the Loess Plateau of China. J. Hydrology 519, 523–531. doi: 10.1016/j.jhydrol.2014.07.043

[B30] Jiang P. Chen N. Zhang X. Yan H. Chen Y. Fan S. (2023). Functional traits and its variation linked to species’ degree of isohydry in subtropical regions with high heterogeneity. Plant Soil 482, 277–296. doi: 10.1007/s11104-022-05688-8

[B31] Jiang Z. Ma K. Liu H. Tang Z. (2018b). A trait-based approach reveals the importance of biotic filter for elevational herb richness pattern. J. Biogeogr. 45, 2288–2298. doi: 10.1111/jbi.13398

[B32] Jiang P. Meinzer F. C. Wang H. Kou L. Dai X. Fu X. (2020a). Below-ground determinants and ecological implications of shrub species’ degree of isohydry in subtropical pine plantations. New Phytol. 226, 1656–1666. doi: 10.1111/nph.16502 32096212

[B33] Jiang P. Wang H. Fu X. Dai X. Kou L. Wang J. (2018a). Elaborate differences between trees and understory plants in the deployment of fine roots. Plant Soil 431, 433–447. doi: 10.1007/s11104-018-3778-3

[B34] Jiang P. Wang H. Meinzer F. C. Kou L. Dai X. Fu X. (2020b). Linking reliance on deep soil water to resource economy strategies and abundance among coexisting understorey shrub species in subtropical pine plantations. New Phytol. 225, 222–233. doi: 10.1111/nph.16027 31247133

[B35] Jobbagy E. Jackson R. B. (2001). The distribution of soil nutrients with depth: Global patterns and the imprint of plants. Biogeochemistry 53, 51–77. doi: 10.2307/1469627

[B36] Jobbagy E. G. Jackson R. B. (2004). The uplift of soil nutrients by plants: biogeochemical consequences across scales. Ecology 85, 2380–2389. doi: 10.2307/3450236

[B37] Klinka K. Green R. N. Trowbridge R. L.. (1981). Taxonomic classification of humus forms in ecosystems of British Columbia: first approximation. Land Management Report 8. J. Ecol. 70, 566–575. doi: 10.2307/2259944

[B38] Kong F. Yu R. Xu Z. Zhou M. (2012). Application of excel in calculation of biodiversity indices. Mar. Sci. 36, 57–62.

[B39] Ksenzhek O. S. Volkov A. G. (1998). Plant energetics (Elsevier Inc.). New York, USA: Academic Press 243–266. doi: 10.1016/B978-012427350-4/50012-1

[B40] Le Roux X. Bariac T. Mariotti A. (1995). Spatial partitioning of the soil water resource between grass and shrub components in a West African humid savanna. Oecologia 104, 147–155. doi: 10.1007/BF00328579 28307351

[B41] Li H. Liu B. McCormack M. L. Ma M. Guo D. (2017). Diverse belowground resource strategies underlie plant species coexistence and spatial distribution in three grasslands along a precipitation gradient. New Phytol. 216, 1140–1150. doi: 10.1111/nph.14710 28758691

[B42] Li B. Zhang W. Li S. Wang J. Liu G. Xu M. (2021). Severe depletion of available deep soil water induced by revegetation on the arid and semiarid Loess Plateau. For. Ecol. Manage. 491, 119156. doi: 10.1016/j.foreco.2021.119156

[B43] Liu Y. Gao G. Wang D. Jiao L. Li Z. Tian L.. (2023). Water use characteristics of *Robinia pseudoacacia* plantations under different thinning intensities in the loess hilly region. Acta Ecol. Sin 303, 2845–2855. doi: 10.5846/stxb202204221107

[B44] Liu B. Li H. Zhu B. Koide R. T. Eissenstat D. M. Guo D. (2015). Complementarity in nutrient foraging strategies of absorptive fine roots and arbuscular mycorrhizal fungi across 14 coexisting subtropical tree species. New Phytol. 208, 125–136. doi: 10.1111/nph.13434 25925733

[B45] Love D. Venturas M. Sperry J. Brooks P. Pettit J. L. Wang Y.. (2019). Dependence of aspen stands on a subsurface water subsidy: Implications for climate change impacts. Water Resour. Res. 55, 1833–1848. doi: 10.1029/2018WR023468

[B46] Luo Y.-H. Cadotte M. W. Burgess K. S. Liu J. Tan S.-L. Xu K.. (2019). Forest community assembly is driven by different strata-dependent mechanisms along an elevational gradient. J. Biogeogr. 46, 2174–2187. doi: 10.1111/jbi.13669

[B47] Maestre F. T. Bautista S. Cortina J. (2003). Positive, negative, and net effects in grass-shrub interactions in mediterranean semiarid grasslands. Ecology 84, 3186–3197. doi: 10.1890/02-0635

[B48] Matjaž Č. Primož S. (2010). Root distribution of under-planted European beech (*Fagus sylvatica* L.) below the canopy of a mature Norway spruce stand as a function of light. Eur. J. For. Res. 129 (4), 531–539. doi: 10.1007/s10342-009-0352-9

[B49] McElrone A. J. Pockman W. T. Martínez-Vilalta J. Jackson R. B. (2004). Variation in xylem structure and function in stems and roots of trees to 20 m depth. New Phytol. 163, 507–517. doi: 10.2307/1514454 33873731

[B50] McIvor I. R. Sloan S. Pigem L. R. (2014). Genetic and environmental influences on root development in cuttings of selected Salix and Populus clones–a greenhouse experiment. Plant Soil 377, 25–42. doi: 10.1007/s11104-013-1770-5

[B51] Meinzer F. C. Andrade J. L. Goldstein G. Holbrook M. N. Cavelier J. Wright S. J. (1999). Partitioning of soil water among canopy trees in a seasonally dry tropical forest. Oecologia 121, 293–301. doi: 10.1007/s004420050931 28308316

[B52] Mi M. Shao M. Liu B. (2016). Effect of rock fragments content on water consumption, biomass and water-use efficiency of plants under different water conditions. Ecol. Eng. 94, 574–582. doi: 10.1016/j.ecoleng.2016.06.044

[B53] Miyatani K. Mizusawa Y. Okada K. Tanikawa T. Makita N. Hirano Y. (2016). Fine root traits in *Chamaecyparis obtusa* forest soils with different acid buffering capacities. Trees-Struct. Funct. 30, 415–429. doi: 10.1007/s00468-015-1291-3

[B54] Moreira M. Z. Sternberg L. D. L. Nepstad D. C. (2000). Vertical patterns of soil water uptake by plants in a primary forest and an abandoned pasture in the eastern Amazon: an isotopic approach. Plant Soil 222, 95–107. doi: 10.1023/A:1004773217189

[B55] Nie Y. P. Chen H. S. Wang K. L. Tan W. Deng P. Y. Yang J. (2011). Seasonal water use patterns of woody species growing on the continuous dolostone outcrops and nearby thin soils in subtropical China. Plant Soil 341, 399–412. doi: 10.1007/s11104-010-0653-2

[B56] Noguchi K. Matsuura Y. Morishita T. Toriyama J. Kim Y. (2021). Fine root growth of black spruce trees and understory plants in a permafrost forest along a north-facing slope in Interior Alaska. Front. Plant Sci. 12. doi: 10.3389/fpls.2021.769710 PMC8635146 34868167

[B57] Novák V. Knava K. (2012). The influence of stoniness and canopy properties on soil water content distribution: simulation of water movement in forest stony soil. Eur. J. For. Res. 131, 1727–1735. doi: 10.1007/s10342-011-0589-y

[B58] Oliva Carrasco L. Bucci S. J. Di Francescantonio D. Lezcano O. A. Campanello P. I. Scholz F. G.. (2015). Water storage dynamics in the main stem of subtropical tree species differing in wood density, growth rate and life history traits. Tree Physiol. 35, 354–365. doi: 10.1093/treephys/tpu087 25428825

[B59] Pabin J. Lipiec J. Wlodek S. Biskupski A. Kaus A. (1998). Critical soil bulk density and strength for pea seedling root growth as related to other soil factors. Soil Till. Res. 46, 203–208. doi: 10.1016/S0167-1987(98)00098-1

[B60] Padilla F. M. Miranda J. D. Pugnaire F. I. (2007). Early root growth plasticity in seedlings of three Mediterranean woody species. Plant Soil 296, 103–113. doi: 10.1007/s11104-007-9294-5

[B61] Pate J. S. Jeschke W. D. Aylward M. J. (1995). Hydraulic architecture and xylem structure of the dimorphic root systems of South-West Australian species of the Proteaceae. J. Exp. Bot. 46, 907–915. doi: 10.1093/jxb/46.8.907

[B62] Pate J. S. Jeschke W. Dawson T. E. Raphael C. Hartung W. Bowen B. J. (1998). Growth and seasonal utilisation of water and nutrients by *Banksia prionotes* . Aust. J. Bot. 46, 511–532. doi: 10.1071/BT97045

[B63] Phillips D. L. Newsome S. D. Gregg J. W. (2005). Combining sources in stable isotope mixing models: alternative methods. Oecologia 144, 520–527. doi: 10.1007/s00442-004-1816-8 15711995

[B64] Pivovaroff A. L. McDowell N. G. Rodrigues T. B. Brodribb T. Cernusak L. A. Choat B.. (2021). Stability of tropical forest tree carbon-water relations in a rainfall exclusion treatment through shifts in effective water uptake depth. Global Change Biol. 27, 6454–6466. doi: 10.1111/gcb.15869 34469040

[B65] Pregitzer K. S. DeForest J. L. Burton A. J. Allen M. F. Ruess R. W. Hendrick R. L. (2002). Fine root architecture of nine North American trees. Ecol. Monogr. 72, 293–309. doi: 10.1890/0012-9615(2002)072[0293:FRAONN]2.0.CO;2

[B66] Prieto I. Roumet C. Cardinael R. Dupraz C. Jourdan C. Kim J. H.. (2015). Root functional parameters along a land-use gradient: evidence of a community-level economics spectrum. J. Ecol. 103, 361–373. doi: 10.1111/1365-2745.12351

[B67] Querejeta J. I. Estrada-Medina H. Allen M. F. Jimenez-Osornio J. J. (2007). Water source partitioning among trees growing on shallow karst soils in a seasonally dry tropical climate. Oecologia 152, 26–36. doi: 10.1007/s00442-006-0629-3 17216213

[B68] Quesada C. A. Hodnett M. G. Breyer L. M. Santos A. J. B. Andrade S. Miranda H. S.. (2008). Seasonal variations in soil water in two woodland savannas of central Brazil with different fire histories. Tree Physiol. 28, 405–415. doi: 10.1093/treephys/28.3.405 18171664

[B69] Ripullone F. Camarero J. J. Colangelo M. Voltas J. (2020). Variation in the access to deep soil water pools explains tree-to-tree differences in drought-triggered dieback of Mediterranean oaks. Tree Physiol. 40, 591–604. doi: 10.1093/treephys/tpaa026 32159804

[B70] Rolo V. Moreno G. (2012). Interspecific competition induces asymmetrical rooting profile adjustments in shrub-encroached open oak woodlands. Trees 26, 997–1006. doi: 10.1007/s00468-012-0677-8

[B71] Romero-Aranda R. Soria T. Cuartero J. (2001). Tomato plant-water uptake and plant-water relationships under saline growth conditions. Plant Sci. 160, 265–272. doi: 10.1016/S0168-9452(00)00388-5 11164598

[B72] Schall P. Lödige C. Beck M. Ammer C. (2012). Biomass allocation to roots and shoots is more sensitive to shade and drought in European beech than in Norway spruce seedlings. For. Ecol. Manage. 266, 246–253. doi: 10.1016/j.foreco.2011.11.017

[B73] Schwartz M. W. Brigham C. A. Hoeksema J. D. Lyons K. G. Mills M. H. van Mantgem P. J. (2000). Linking biodiversity to ecosystem function: implications for conservation ecology. Oecologia 122, 297–305. doi: 10.1007/s004420050035 28308280

[B74] Schwinning S. (2010). The ecohydrology of roots in rocks. Ecohydrology 3, 238–245. doi: 10.1002/eco.134

[B75] Šenfeldr M. Urban J. Maděra P. Kučera J. (2016). Redistribution of water via layering branches between connected parent and daughter trees in Norway spruce clonal groups. Trees 30, 5–17. doi: 10.1007/s00468-015-1157-8

[B76] Strock C. F. Burridge J. D. Niemiec M. D. Brown K. M. Lynch J. P. (2021). Root metaxylem and architecture phenotypes integrate to regulate water use under drought stress. Plant Cell Environ. 44, 49–67. doi: 10.1111/pce.13875 32839986

[B77] Sun T. Dong L. Mao Z. Li Y. (2015). Fine root dynamics of trees and understorey vegetation in a chronosequence of *Betula platyphylla* stands. For. Ecol. Manage. 346, 1–9. doi: 10.1016/j.foreco.2015.02.035

[B78] Szota C. Veneklaas E. J. Koch J. M. Lambers H. (2007). Root architecture of jarrah (*Eucalyptus marginata*) trees in relation to post-mining deep ripping in Western Australia. Restor. Ecol. 15, 65–73. doi: 10.1111/j.1526-100X.2007.00294.x

[B79] Tetegan M. Korboulewsky N. Bouthier A. Samouëlian A. Cousin I. (2015). The role of pebbles in the water dynamics of a stony soil cultivated with young poplars. Plant Soil 391, 307–320. doi: 10.1007/s11104-015-2429-1

[B80] Vadez V. (2014). Root hydraulics: the forgotten side of roots in drought adaptation. Field Crops Res. 165, 15–24. doi: 10.1016/j.fcr.2014.03.017

[B81] Valladares F. Pearcy R. W. (2002). Drought can be more critical in the shade than in the sun: a field study of carbon gain and photo-inhibition in a Californian shrub during a dry El Nino year. Plant Cell Environ. 25, 749–759. doi: 10.1046/j.1365-3040.2002.00856.x

[B82] Walker B. H. Noy-Meir I. (1982). Aspects of the stability and resilience of savanna ecosystems. In Ecology of Tropical Savannas. Ecol. Stud. 42, 556–590. doi: 10.1007/978-3-642-68786-0

[B83] Wang J. Fu B. J. Lu N. Zhang L. (2017). Seasonal variation in water uptake patterns of three plant species based on stable isotopes in the semi-arid Loess Plateau. Sci. Total Environ. 609, 27–37. doi: 10.1016/j.scitotenv.2017.07.133 28734247

[B84] Wen X. Lee X. Sun X. Wang J. Hu Z. Li S.. (2012). Dew water isotopic ratios and their relationships to ecosystem water pools and fluxes in a cropland and a grassland in China. Oecologia 168, 549–561. doi: 10.1007/s00442-011-2091-0 21822725

[B85] West A. G. Hultine K. R. Burtch K. G. Ehleringer J. R. (2007). Seasonal variations in moisture use in a pinion-juniper woodland. Oecologia 153, 787–798. doi: 10.1007/s00442-007-0777-0 17576601

[B86] Wu Z. Behzad H. M. He Q. Wu C. Bai Y. Jiang Y. (2021). Seasonal transpiration dynamics of evergreen Ligustrum lucidum linked with water source and water-use strategy in a limestone karst area, southwest China. J. Hydrol. 597, 126199. doi: 10.1016/j.jhydrol.2021.126199

[B87] Wu X. Niu Y. Xun M. Jin J. Li N. Tang Y.. (2022). Responses of water source to seasonal drought of dominant afforestation tree species in the loess hilly region of China. Acta Ecol. Si. 42, 4101–4112. doi: 10.5846/stxb202106221657

[B88] Yang B. Wen X. Sun X. (2015). Seasonal variations in depth of water uptake for a subtropical coniferous plantation subjected to drought in an East Asian monsoon region. Agr. For. Meteorol. 201, 218–228. doi: 10.1016/j.agrformet.2014.11.020

[B89] Yang F. Feng Z. Wang H. Dai X. Fu X. (2017). Deep soil waterextraction helps to drought avoidance but shallow soil water uptake during dry season controls the inter-annual variation in tree growth in four subtropical plantations. Agr. For. Meteorol 234, 106–114. doi: 10.1016/j.agrformet.2016.12.020

[B90] Yu K. L. D'Odorico P. (2015). Hydraulic lift as a determinant of tree–grass coexistence on savannas. New Phytol. 207, 1038–1051. doi: 10.1111/nph.13431 25925655

[B91] Zhu W. Li W. Shi P. Cao J. Zong N. Geng S. (2021). Intensified interspecific competition for water after afforestation with *Robinia pseudoacacia* into a native shrubland in the Taihang Mountains, Northern China. Sustainability 13, 807. doi: 10.3390/su13020807

