# Supplementary material for: Patterns of deep fine root and water utilization amongst trees, shrubs and herbs in subtropical pine plantations with seasonal droughts
Source: Front Plant Sci. 2023 Sep 20;14:1275464. doi: 10.3389/fpls.2023.1275464 (PMC10548128; doi:10.3389/fpls.2023.1275464)
Supplement: Supplementary file 1 [file DataSheet_1.docx]

Table S1 Relationships between community structure and deep soil water utilization of trees and shrubs in the dry season

|  | Tree_Dry_ | | Shrub_Dry_ | |
| --- | --- | --- | --- | --- |
|  | *r* | *P* | *r* | *P* |
| Dominance of shrubs | 0.481 | 0.008 | 0.320 | 0.091 |
| Richness of shrubs | 0.299 | 0.115 | 0.110 | 0.569 |
| Evenness of shrubs | 0.316 | 0.095 | 0.231 | 0.227 |
| Dominance of herbs | 0.040 | 0.839 | 0.204 | 0.288 |
| Richness of herbs | 0.183 | 0.343 | 0.455 | 0.013 |
| Evenness of herbs | -0.089 | 0.646 | -0.128 | 0.508 |

Tree_Dry_, deep soil water utilization of trees in the dry season; Shrub_Dry_, deep soil water utilization of trees in the dry season.


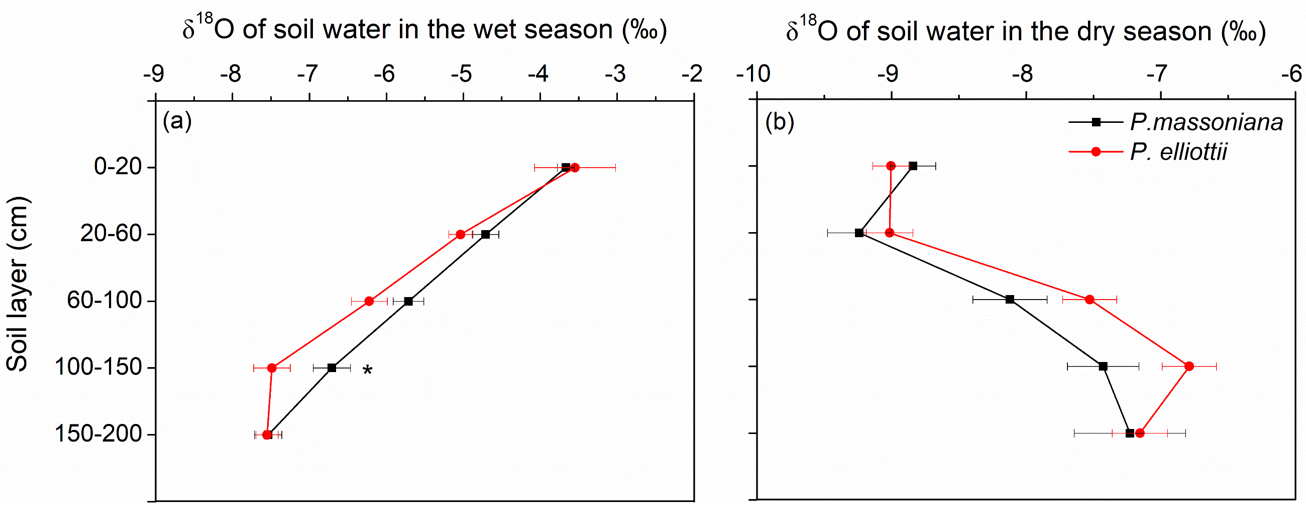


Fig. S1 Vertical distribution of soil water δ^18^O between seasons in the *P. massoniana* and *P. elliottii* forests. * indicate significant differences between *P. massoniana* and *P. elliottii* forests.


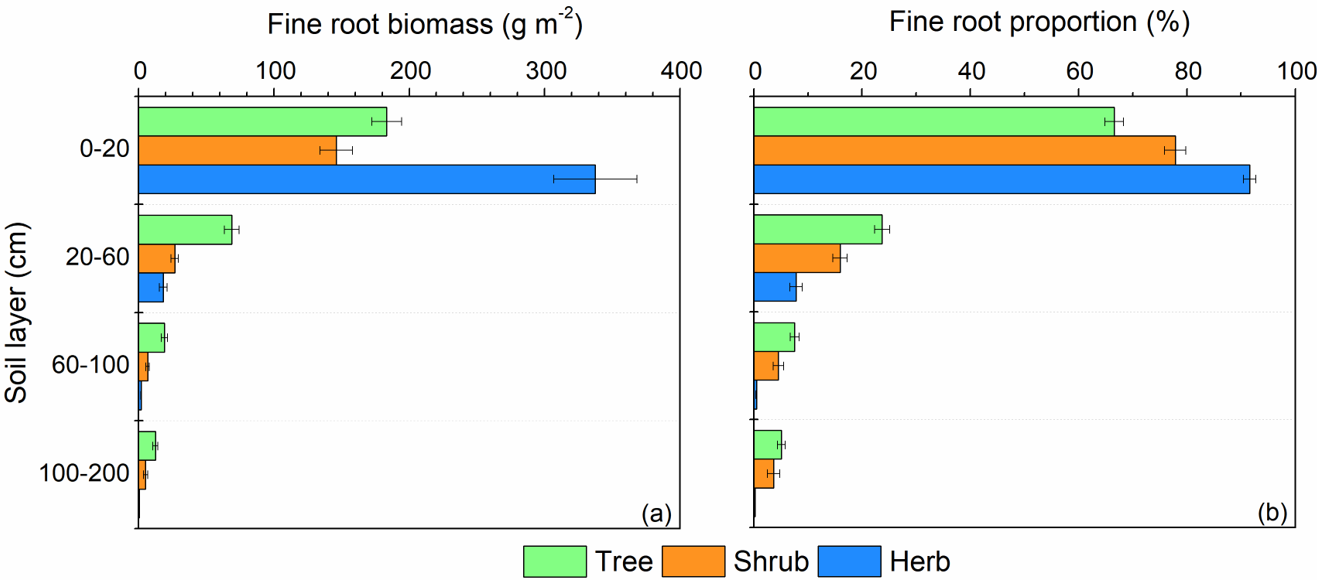


Fig. S2 Fine root biomass and its proportion of different soil layer amongst life forms


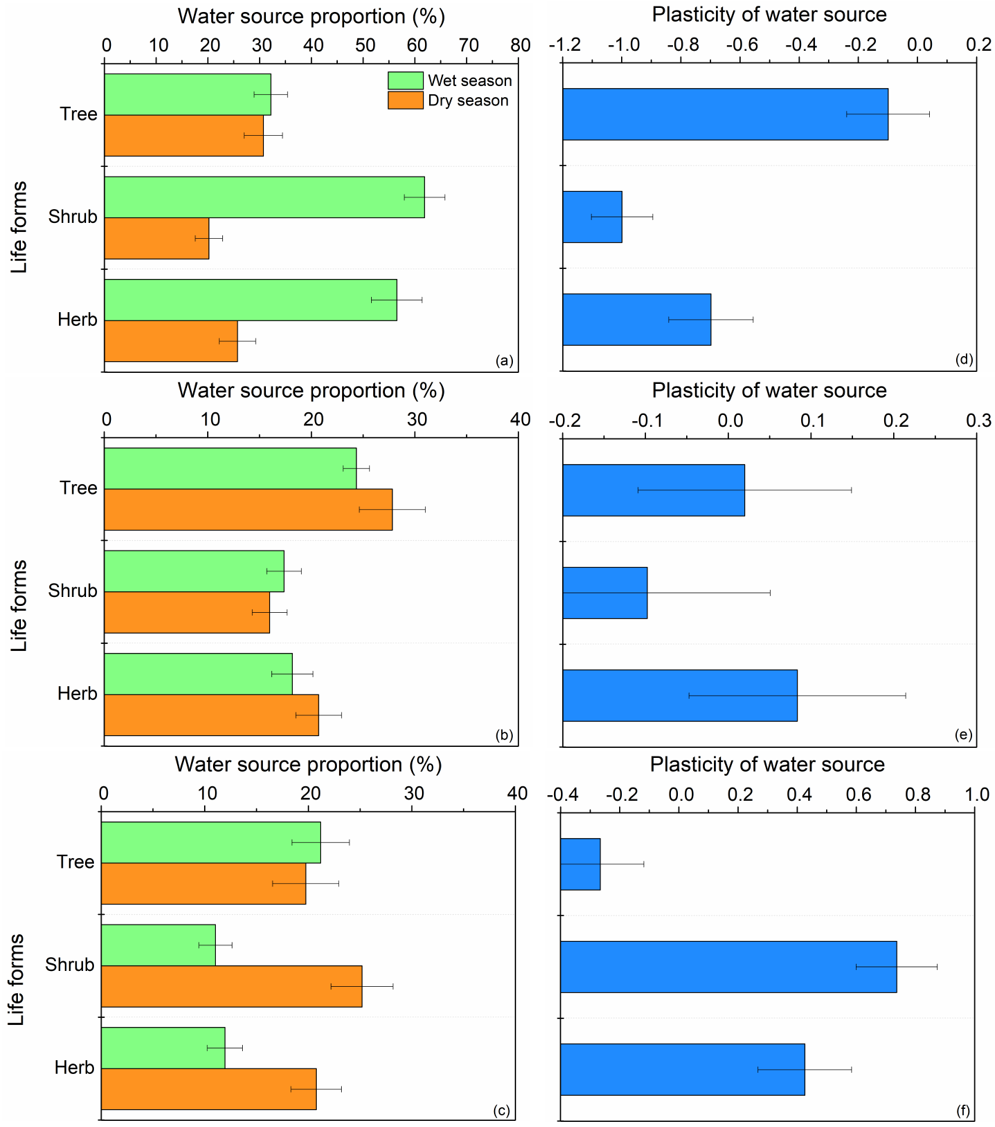


Fig. S3 Water source proportion of 0-20, 20-60, 60-100 cm soil layer in the wet and dry season (a-c) and their plasticity between seasons (d-f) amongst trees, shrubs and herbs.
